# Hybrid Window Decoding for Joint Source Channel Anytime Coding System

**DOI:** 10.3390/e26110940

**Published:** 2024-11-01

**Authors:** Li Deng, Zhiping Shi, Yixin Wang, Xiaoxi Yu, Yong Liang Guan, Zhiping Xu

**Affiliations:** 1 National Key Laboratory on Communications, University of Electronic Science and Technology of China, Chengdu 611731, China; dengli@uestc.edu.cn; 2 Yangtze Delta Region Institute (Huzhou), University of Electronic Science and Technology of China, Huzhou 313000, China; 3 The Institute for Infocomm Research (I2R), Agency for Science, Technology and Research (A*STAR), Singapore 138632, Singapore; wang_yixin@i2r.a-star.edu.sg; 4 The School of Electrical and Electronic Engineering, Nanyang Technological University, Singapore 639798, Singapore; xiaoxi001@e.ntu.edu.sg (X.Y.); eylguan@ntu.edu.sg (Y.L.G.); 5 The School of Ocean Information Engineering, Jimei University, Xiamen 361021, China; zhipingxu@jmu.edu.cn

**Keywords:** joint source channel coding (JSCC), anytime coding, adaptive local expanding window decoding

## Abstract

Joint source channel anytime coding (JSCAC) is a kind of joint source channel coding (JSCC) systems based on the causal spatially coupled coding and joint expanding window decoding (JEWD) techniques. JSCAC demonstrates greatly improved error correction performance, as well as higher decoding complexity. This work proposes a joint hybrid window decoding (JHWD) algorithm for JSCAC systems, aiming to reduce the decoding complexity while maintaining comparable error correction performance with the state of the art. Unlike the traditional JEWD technique and its variants, the proposed JHWD algorithm utilizes a hybrid window structure. It achieves this by implementing adaptive local expanding window decoding in the sliding window region, guided by syndrome-check-based detection. The hybrid window decoding characteristics of the proposed JHWD algorithm can both effectively reduce the decoding complexity caused by window expanding, and also mitigate the error propagation caused by window sliding. An improved density evolution algorithm is proposed for the asymptotic performance analysis of the proposed JHWD algorithm. Both the analytical and simulation results indicate that the proposed JHWD decoding scheme is a good low-complexity implementation option for JSCAC systems, which is also suitable for other JSCC systems with the spatially coupled coding structure.

## 1. Introduction

The separate source-channel coding technique is based on the Shannon separation coding theorem, which assumes that the source entropy is less than the channel capacity under the conditions of unrestricted delay, complexity and code length. That is, the separation coding is asymptotically optimal. Joint source channel coding (JSCC) is a kind of coding technology that designs source/channel encoding and decoding in an interdependent way, so as to achieve the overall optimal communication system [1]. JSCC technology can be used in any system that violates the condition of the separation coding theorem and needs to consider both the source side and the channel side, such as communication systems with limitations of power, bandwidth, complexity or delay, multi-user system with shared channel, and different types of communication environments composed of heterogeneous sources or channels, etc. In recent years, the increasingly mature deep learning technology has brought new design ideas for JSCC technology, and set off a new wave of research [2,3,4].

The benefits of JSCC are reflected in the following aspects: (a) Improve the transmission efficiency: with comprehensive consideration of the characteristics of the source and channel, JSCC can optimize the coding scheme, reduce the size of the transmitted data, so as to improve the transmission efficiency. This means that more information can be transmitted with the same bandwidth, or less transmission time with the same amount of information. (b) Reduce the error rate: JSCC can adjust the coding mode according to the specific situation of the channel to reduce the bit error rate during transmission. This means that data are less error-prone during transmission, improving the reliability and accuracy of data. (c) Improve the system robustness: JSCC is designed with channel variation and interference in mind, making the system more stable and reliable in the face of different channel conditions. This allows the system to adapt to a wider range of environments, reducing transmission failures due to channel changes.

In terms of JSCC technologies that are not based on deep learning, there are several typical schemes. At the sending side, the channel encoder can provide the channel state information (CSI) to the source encoder for the bit allocation [5,6] and the source code optimization [7]. On the other hand, the source encoder can also provide the source significance information (SSI) for the unequal error protection (UEP) during the channel coding or modulation [8,9]. At the receiving side, the source *a prior* information and *a posterior* information can be used for iterative decoding between the source and channel decoders, which is one of the most popular JSCC solutions and also the focus of this paper. Fresia first proposed a JSCC system that completes the source compression and error correction using two block LDPC codes (D-LDPC), then implements the joint belief propagation (BP) decoding within a global factor graph model framework, including both source and channel coding structures [10]. After that, various codes were introduced into the JSCC system, such as the block double protograph LDPC (DP-LDPC) code [11,12], the block double Polar (D-Polar) code [13], the spatially coupled LDPC (SC-LDPC) code [14,15,16], and the spatially coupled repeat-accumulate (SC-RA) code [17,18], etc.

Among the above solutions, we focus on the scheme termed SC-RA based joint source channel anytime coding (JSCAC). It is a specific JSCC scheme combined with anytime coding techniques, i.e., causal spatially coupled coding and expanding window decoding [19,20]. The anytime properties of the JSCAC scheme account for both delay and reliability, making it particularly suitable for transmission scenarios demanding both low delay and high reliability. However, the prior-art expanding window decoding strategies have noticeable decoding complexities and memory space requirements [17,18], which affect the practical applications of JSCAC systems.

In order to reduce the decoding complexity while maintaining comparable error correction performance, a hybrid window decoding scheme is proposed. The novelty and contributions of this paper can be summarized as follows.

- A joint hybrid window decoding (JHWD) algorithm is proposed, which adaptively implements the local expanding window decoding in the sliding window region with the constraint of syndrome-check based detection.
- The density evolution algorithm of the JSCAC system in [18] is improved for the proposed JHWD scheme to analyze its asymptotic decoding complexity and anytime properties.
- Some numerical analyses and simulation results are provided to confirm the advantages of the proposed JHWD algorithm over other prior-art decoding schemes for JSCAC systems.

The remainder of this paper is organized as follows. The background of the SC-RA-based JSCAC system is briefly introduced in Section 2. The specific descriptions of the proposed JHWD scheme and improved density evolution algorithm are provided in Section 3. The numerical analyses and simulation results are presented in Section 4, followed by conclusions in Section 5.

## 2. Preliminaries

In this section, we briefly introduce the coding structure, the prior-art decoding strategies, and the density evolution analysis method of SC-RA-based JSCAC system.

### 2.1. Source Model

Let $s=\left(s_{1},\cdots ,s_{2}\right)\in {\left\{0,1\right\}}^{n}$ be a binary sequence of length *n* generated by an independent identically distributed (i.i.d) nonuniform memory-less source with probability distribution (p,1−p), where *p* is the probability of the binary bit “1” in a source sequence (named as source probability), and $p=P_{r}\left(s_{i}=1\right)\neq 0.5$. The initial log-likelihood-ratio (LLR) of each source bit is denoted by $z^{sc}=\log\left(\frac{\left(1-p\right)}{p}\right)$; and the initial LLRs of the received code word y after the binary-phase-shift-keying modulation (BPSK) and over the additive white Gaussian noise (AWGN) channel can be represented as $z^{cc}=\frac{2y}{\sigma^{2}}$, where $\sigma^{2}$ is the variance of channel noise.

### 2.2. Sketch of the SC-RA-Based JSCAC System

**Figure 1:**
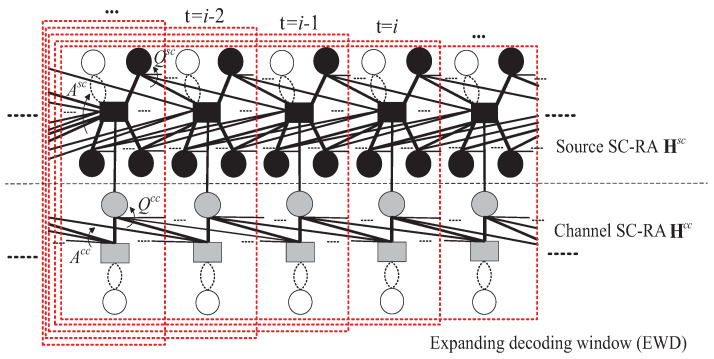
Encoding and decoding structures of the SC-RA-based JSCAC system [17]. Reproduced with permission of [17], *Copyright* ©2020, *IEEE*.

Figure 1 shows the encoding and decoding structures of the SC-RA-based JSCAC system [17], which is composed of the upper half part (source code) and the lower half part (channel code). Therein, the white circles represent the parity bit nodes (PNs) with a fixed degree of 2; the black and gray rectangles indicate the source syndrome nodes (SNs) and channel check nodes (CNs) with degrees of $A^{sc}$ and $A^{cc}$, respectively; while the black and gray circles denote the source and channel information bit nodes (INs) with degrees of $Q^{sc}$ and $Q^{cc}$, respectively. Each SN or CN is causally and exponentially connected to the former INs with an exponential rate of λ and a coupling length of γ. The exponentially distributed edge connections of source code and channel code are depicted by line thickness in Figure 1, which can also be written as [21]:

(1) $$P_{r}\left(k\right)=\frac{e^{-k\lambda }\left(1-e^{-\lambda }\right)}{1-e^{-\gamma \lambda }},$$

where k=j−i is the distance between the *j*-th SN (CN) with the connected *i*-th IN (j≥i). The exponential rate of $\lambda =\lambda^{sc}$ (or $\lambda^{cc}$) and the coupling length of $\gamma =\gamma^{sc}$ (or $\gamma^{cc}$). The larger the parameter λ, the faster the error probability decays with increasing decoding delay. When $\lambda^{sc}=\lambda^{cc}=0$, then $P_{r}\left(k\right)=1$, which is equal to the uniform distribution. The uniform distribution has a slow decline rate over the decoding delay, which is undesired in the anytime transmission. The source code rate and channel code rate can be calculated with the following equation [22]:

(2) $$R=\frac{L}{L+\frac{Q}{A}\left(L-\gamma +1+2\left(\gamma -\sum_{i=0}^{\gamma }{\left(\frac{i}{\gamma }\right)}^{A}\right)\right)},$$

where *L* is the chain length. For the source code rate $R^{sc}$, $Q=Q^{sc}$, $A=A^{sc}$, $\gamma =\gamma^{sc}$; and for the channel code rate $R^{cc}$, $Q=Q^{cc}$, $A=A^{cc}$, $\gamma =\gamma^{cc}$. As L→∞, the asymptotic code rates of the SC-RA-based JSCAC system are $R^{cc}\to \frac{A^{cc}}{A^{cc}+Q^{cc}}$, $R^{sc}\to \frac{Q^{sc}}{A^{sc}+Q^{sc}}$.

### 2.3. Encoding Procedure of SC-RA-Based JSCAC System

The source parity-check matrix $H^{sc}$ and the channel parity-check matrix $H^{cc}$ have the same structure as (3)

(3) $$H_{\left[0,L-1\right]}=\left[\begin{array}{ccc} H_{00} & \; & \\ \vdots & \ddots & \\ H_{0(\gamma -1)} & \ddots & \\ \; & \ddots & H_{(L-1)0}\\ \; & \; & \vdots \\ \; & \; & H_{\left(L-1)(\gamma -1\right)} \end{array}\right],$$

where *L* is the chain length, the involved sub-matrices for the encoding procedure of the *i*-th message block are from $H_{i0}$ to $H_{\left(i-\gamma +1)(\gamma -1\right)}$, $\gamma =\gamma^{sc}$ or ($\gamma^{cc}$). The proposed JSCAC follows the concatenated source and channel encoding of block LDPC based JSCC systems.

(1) Syndrome source encoding: Assume the causal source streaming $s_{\left[0,L-1\right]}=\left\{s_{i}|0\leq i\leq L-1\right\}$ produces a $N^{sc}$-bit message block $s_{i}$ at a time instant *t* and with a chain length of *L*. The message block $s_{i}$ is compressed with the syndrome source coding as $u_{i}=s_{i}H_{i0}^{{sc}^{T}}+s_{\left(i-1\right)}H_{(i-1)1}^{{sc}^{T}}+\ldots +s_{\left(i-\gamma +1\right)}H_{\left(i-\gamma +1)(\gamma -1\right)}^{{sc}^{T}}$ with the block-length of $M^{sc}$. If i<γ−1, then $u_{i}=s_{i}H_{i0}^{{sc}^{T}}+s_{\left(i-1\right)}H_{(i-1)1}^{{sc}^{T}}+\ldots +s_{0}H_{0(i-1)}^{{sc}^{T}}$.

(2) Channel encoding: The compressed source sequence $u_{\left[0,L-1\right]}=\left\{u_{i}|0\leq i\leq L-1\right\}$ is encoded with the channel encoder to generate the channel codeword $x_{\left[0,L-1\right]}=\left\{x_{i}|0\leq i\leq L-1\right\}$. For each block $x_{i}=\left[u_{i},c_{i}\right]$ with the block-length of $N^{cc}$, the channel PNs $c_{i}$ have a fixed degree of 2, checking both the coupled INs in the early transmitted blocks and local INs, which are denoted as $v_{i}=\left[x_{i-\gamma +1},\ldots ,x_{i-1},u_{i}\right]$ ($v_{i}=\left[x_{0},\ldots ,x_{i-1},u_{i}\right]$, if i<γ−1). The generation of channel PNs $c_{i}=\left\{c_{ij}|1\leq j\leq N^{cc}-M^{sc}\right\}$ is described as

(4) $$c_{ij}=\left\{\begin{array}{cc} v_{i}{\tilde{H}}^{{cc}^{T}}\left(1,:\right), & j\;=\;1\\ v_{i}{\tilde{H}}^{{cc}^{T}}\left(j,:\right)+c_{i(j-1)}, & \mathrm{otherwise} \end{array}\right.,$$

where ${\tilde{H}}^{cc}=\left[H_{\left(i-\gamma +1)(\gamma -1\right)}^{cc},\ldots ,H_{(i-1)1}^{cc},H_{i0}^{cc}\left(:,1:M^{sc}\right)\right]$.

### 2.4. Decoding Algorithms of SC-RA-Based JSCAC System

The red dashed rectangles in Figure 1 indicate the decoding windows expanding in size as decoding delay increases. The joint BP decoding for D-LDPC based JSCC [10] is implemented in each decoding window, which performs the source BP decoder and channel BP decoder in parallel, exchanging the mutual information through the public edges (red dashed lines in Figure 1). See [10] for more details of joint BP. This classical decoding method for JSCAC systems is the joint expanding window decoding (JEWD) [17], which directly implements the joint BP decoding in the expanding window with high complexities. Authors in [17] proposed a partial joint expanding window decoding (PJEWD) based on the observation of convergence inconsistency between the source and channel decoders, i.e., stop message updating for all channel nodes once the channel decoding convergence is detected. Compared with the JEWD algorithm, the PJEWD algorithm can improve the error performance. However, the reduction ratio of decoding complexity is not significant, as it is affected by the channel conditions.

### 2.5. Density Evolution (DE) Algorithms for SC-RA-Based JSCAC System

In [18], the authors proposed a density evolution (DE) algorithm for an SC-RA-based JSCAC system with PJEWD scheme. Based on the DE analysis, the anytime properties of the SC-RA-based JSCAC system are provided, i.e., the error probability of any message block can exponentially decay to zero as the decoding delay approaches to infinity. Moreover, the lower bound of the delay exponent α is also provided, which is proportional to the product of $\lambda^{sc}$ and $Q^{sc}$. The DE algorithm can also analyze the BP decoding threshold and the decoding complexity of the SC-RA-based JSCAC with PJEWD scheme. See more details of the encoding process, the decoding algorithms, and the DE algorithm of the SC-RA-based JSCAC system in [17,18].

## 3. Hybrid Window Decoding for JSCAC System

### 3.1. Hybrid Window Decoding Structure

In this section, we first describe the proposed JHWD algorithm for JSCAC systems in detail, then we provide the improved DE analysis method for the proposed JHWD algorithm.

**Figure 2:**
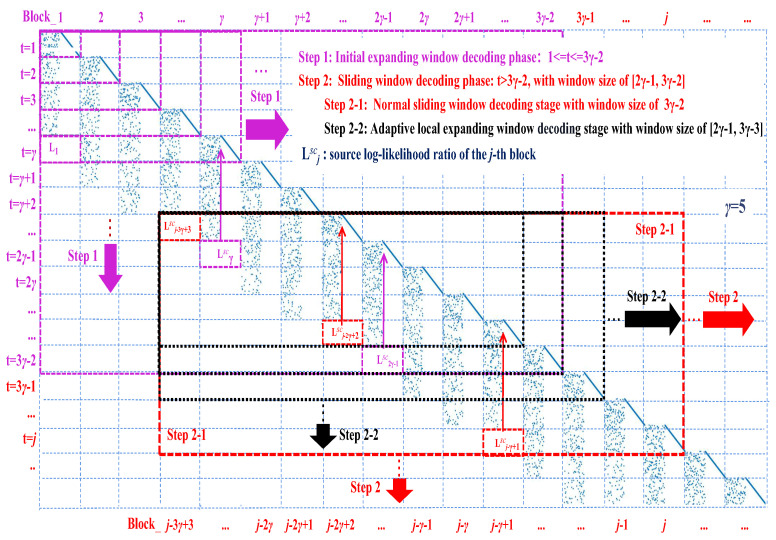
Hybrid window decoding structure of the proposed JHWD algorithm.

The proposed hybrid window decoding structure is shown in Figure 2, which is composed of two main parts, i.e., the initial expanding window decoding phase and the sliding window decoding phase. Each part is numerically marked. In order to simplify the description, the source and channel couple lengths are set the same as $\gamma^{sc}=\gamma^{cc}=\gamma$. In the example shown in Figure 2, γ is set as 5. We explain the details of the proposed JHWD scheme as follows.

*Application scenarios*: The proposed JHWD scheme is designed for the source streaming with short block length, and with a chain length *L* far longer than the coupling length γ, i.e., L≫γ. Accordingly, as shown in Figure 2, there is not any special decoding process for blocks at the end of the data chain. Moreover, this work focuses on the decoding performances for the source streaming with short block lengths in low-latency and high-reliability communications.

*Selection of window size*: In the JSCAC system, the coupling length γ is supposed to be finite, and the encoding structure of any block is directly related to its γ−1 coupled blocks. However, all blocks in the data chain are causally coupled and connected one by one. Therefore, the decoding performance of any block is still related to the backward-connected blocks beyond the coupling length. However, the mutual influence will gradually weaken as distance increases. In order to trade off the error correction performance and the decoding complexity, we adopt the sliding decoding window size as 3γ−2 block lengths, covering the coupling range where the object block is located and the two nearest adjacent coupling ranges with the greatest influences. These are called the first, the second and the third coupling interval, respectively.

*Emendatory decoding strategies*: Combined with the encoding structure of JSCAC system, some emendatory decoding strategies are adopted in the proposed JHWD scheme.

(1) Update the source *a posteriori* log-likelihood ratios (LLRs) at each time slot *t* after decoding as follows

(5) $$L_{t}^{sc}=\left(L_{t-1}^{sc}+L_{t}^{sc}\right)/2.$$

The reason for updating the source LLRs instead of the channel LLRs is that the source sequence is not actually transmitted through the channel; rather, it is represented by its compressed syndrome. It is found that decoding the object block with the previously well-decoded source information can effectively improve the error correction performance.

(2) In order to alleviate the error propagation caused by sliding window decoding [23], a kind of syndrome-check based detection, shown in (6), is carried out before the local expanding window decoding, where ${\hat{S}}_{k}^{sc}$ and ${\hat{U}}_{k}^{cc}$ are the source and channel decoding results of *k*-th joint BP, and ${\hat{H}}_{k}^{sc}$ is the involved source parity-check matrix. If the detection result is false, a local expanding window decoding is adaptively carried out within the decoding window until the detection result is true. This syndrome-check-based detection is implemented once, prior to the matrix multiplication in the joint BP decoding stage of the local expanding window decoding. However, it controls the adaptive execution of the local expanding window decoding, and thus can greatly reduce the decoding iterations and message updates.

(6) $$S_{check0}=\left\{\begin{array}{cc} 1 & \;\mathrm{if}\;\;\;\;\;{\hat{S}}_{k}^{sc}\;\cdot \;{\hat{H}}_{k}^{\mathrm{sc}}={\hat{U}}_{k}^{cc},\\ 0 & else. \end{array}\right.$$

*Decoding structure descriptions*: The details of the JHWD algorithm are provided in Algorithm 1. The parameters $H^{sc}$ and $H^{cc}$ indicate the source and channel parity-check matrix. $I_{\max1}$ and $I_{\max2}$ denote the maximum iterations of different decoding stages; and $I_{\max2}$ is far smaller than $I_{\max1}$. *p* is the source probability, $\sigma_{ch}$ is the variance of channel noise, and *T* is the maximum observed decoding delay.

There are two main steps in Algorithm 1. First, in the initial expanding window decoding phase of Step 1, the traditional JEWD in [17] is implemented within the time slots range of t∈[1,3γ−2]. Second, when t>3γ−2, the window is slid forward one block and the sliding window decoding is implemented at each time slot in Step 2.

Especially, in the sliding window decoding phase of Step 2, it also includes two sub-steps. (a) Step 2-1: In the normal sliding window decoding stage, the initial source LLR, i.e., $z^{sc}=log\frac{1-p}{p}$, is used for decoding with $I_{\max1}$, and the decoding range is the whole window length, i.e., 3γ−2. (b) Step 2-2: In the adaptive local expanding window decoding stage, the source updated *a posteriori* LLRs $L_{t}^{sc}$ is used for decoding with $I_{\max2}$. The decoding range expands with the window size region of [2γ−1,3γ−3], including the first 2γ+k−2 blocks (1≤k<γ) of the sliding window. The syndrome-check-based detection is carried out before the local expanding window decoding. As shown in line 16 to line 24 of Algorithm 1, if $S_{check0}=0$, the local expanding window decoding is carried out within the sliding window region. If $S_{check0}=1$, the adaptive local expanding window decoding is terminated, and the *a posterior* source LLRs are updated for the involved blocks with (5). After each local expanding window decoding, we update $L_{t}^{sc}$ only for blocks in the first coupling interval. The motivation is that in any decoding window range, the LLRs of message blocks in the first coupling interval can converge earlier than the blocks in the other two coupling intervals.
**Algorithm 1:** Joint hybrid window decoding (JHWD) algorithm
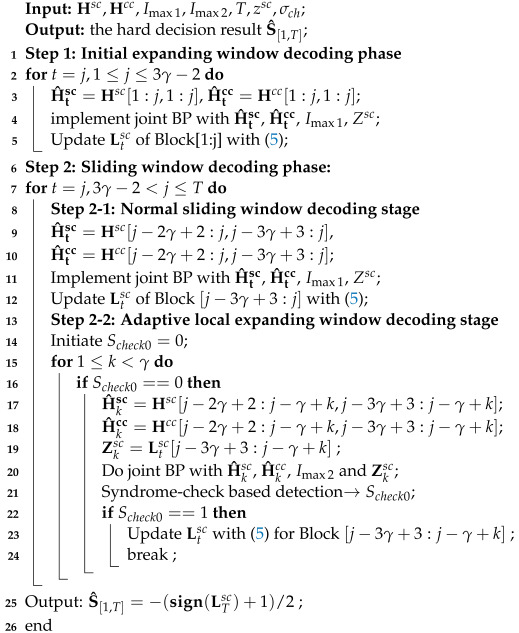


### 3.2. Density Evolution Analysis for JHWD Algorithm

In order to analyze the anytime properties and decoding complexity for the JHWD algorithm, we revised the density evolution algorithm for JSCAC with PJEWD scheme in [18]. In the following density evolution analyses, the specific formulas for message updates are omitted, which are the same as (6) to (15) in [18], and are referred to as “***Message updates***” for simplicity of description.

**Step 1**: Initialize the parameters of $x_{cv}^{0}\left(i_{c},t\right)=x_{cp}^{0}\left(j_{c},t\right)=\mu_{0}=\frac{2}{\sigma_{n}^{2}}$, $0<i_{c},j_{c}\leq t$, and $x_{sv}^{0}\left(i_{s},t\right)=x_{sp}^{0}\left(j_{s},t\right)=\nu_{0}=\log\frac{1-p}{p}$, $0<i_{s},j_{s}\leq t$, where $x_{cv}^{l}(i_{c},t$) and $x_{cp}^{l}\left(i_{c},t\right)$ are messages exchanged from channel IN_*i*_*c*__ and channel PN_*j*_*c*__ to connected channel CNs at the *l*-th iteration and time *t*; $x_{sv}^{l}\left(i_{s},t\right)$ ($x_{sp}^{l}\left(j_{s},t\right)$) are messages exchanged from source IN_*i*_*s*__ (source PN_*j*_*s*__) to connected source SNs at the *l*-th iteration and time *t*. Initialize the number of message updates as $Num_{me}=0$, and the number of iterations as l=0.

**Step 2**: Initial expanding window decoding (1<t≤3γ−2)

(1) Implement “***Message updates***” at the time slot *t* within the expanding window of [1:t] with $I_{\max1}$.

(2) Update the following messages as follows:

$$x_{cv}^{t}\left(i_{c},t\right)=\left(x_{cv}^{t-1}\left(i_{c},t\right)+x_{cv}^{t}\left(i_{c},t\right)\right)/2,$$

$$x_{cp}^{t}\left(j_{c},t\right)=\left(x_{cp}^{t-1}\left(j_{c},t\right)+x_{cp}^{t}\left(j_{c},t\right)\right)/2,$$

$$x_{sv}^{t}\left(i_{s},t\right)=\left(x_{sv}^{t-1}\left(i_{s},t\right)+x_{sv}^{t}\left(i_{s},t\right)\right)/2,$$

(7) $$x_{sp}^{t}\left(j_{s},t\right)=\left(x_{sp}^{t-1}\left(j_{s},t\right)+x_{sp}^{t}\left(j_{s},t\right)\right)/2.$$

(3) Update the number of message updates $Num_{me}$.

**Step 3**: Sliding window decoding (3γ−2<t≤T)

(1) Normal sliding window decoding stage: implement “***Message updates***” at the time slot *t* within the sliding window of [t−3γ+3:t] with $I_{\max1}$; then update messages with (7), and update $Num_{me}$.

(2) Adaptive local expanding window decoding stage: The syndrome-check-based detection is adopted by checking $x_{sv}^{t}\left(i_{s},t\right)$, as below:

(8) $$S_{check1}=\left\{\begin{array}{cc} 1 & \;\mathrm{if}\;\;\;\;\;|x_{sv}^{t}\left(i_{s},t\right)-x_{sv}^{t-1}\left(i_{s},t\right)|<\epsilon ,\\ 0 & else. \end{array}\right.$$

where ϵ is a threshold with a very small value. If $S_{check1}=0$, implement “***Message updates***” at the time slot *t* within the local expanding window of [t−3γ+3:t−γ+k] (k=2:γ) with $I_{\max2}$. If $S_{check1}=1$, which means that $x_{sv}^{t}\left(i_{s},t\right)$ could not update anymore compared with $x_{sv}^{t-1}\left(i_{s},t\right)$, update messages with (7) and $Num_{me}$.

**Step 4**: Calculate the error probability with (17) in [18].

## 4. Simulation and Analysis

In this section, we explore the decoding complexity, anytime properties and error correction performance of the proposed JHWD algorithm with both numerical analyses and simulations. The benchmark algorithms are the joint expanding window decoding (JEWD) algorithm and the partial joint expanding window decoding (PJEWD) algorithm for JSCAC systems in [17,18]. We also compared the proposed algorithm with the traditional joint source channel decoding (JSCD) for block D-LDPC-based JSCC systems [10] and the SC-LDPC-based JSCC scheme with sliding variable-window decoding (SVWD) [16]. The additive white Gaussian noise (AWGN) channel and the binary phase-shift keying (BPSK) modulation are adopted. The simulation parameters are set as follows: $Q^{sc}=4$, $A^{sc}=12$, $Q^{cc}=3$, $A^{cc}=3$, $R^{sc}=0.25$, $R^{cc}=0.5$, $\lambda^{sc}=0.5$, $\lambda^{cc}=0.1$, $I_{\max1}=30$, $I_{\max2}=5$, p=0.02, and $E_{b}/N_{0}$ = 2 dB for anytime properties analyses.

**Figure 3:**
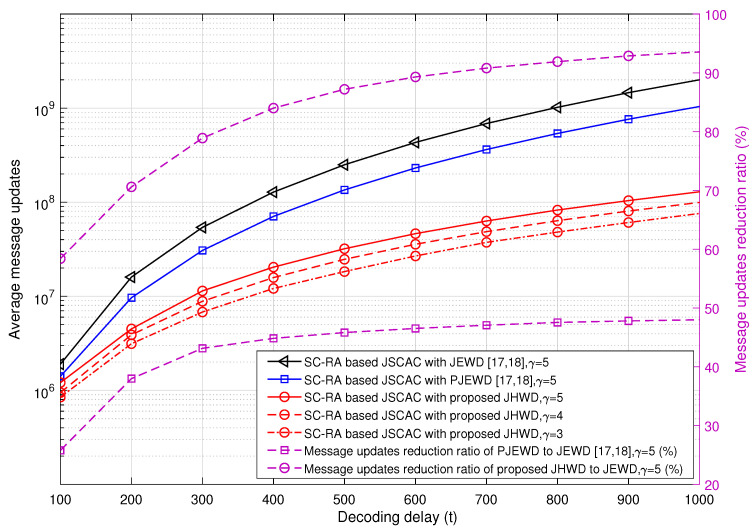
Asymptotic decoding complexity analysis based on the density evolution [17,18].

We first analyze the asymptotic average message updates of the SC-RA-based JSCAC system as the decoding delay using different decoding schemes. As shown in Figure 3, as the decoding delay increases, the average message updates of all the three decoding schemes show slow and nearly linear growth trends, and their differences become more significant. The right Y-axis of Figure 3 shows the average message updates reduction ratio of PJEWD and the proposed JHWD compared to the traditional JEWD algorithm. As *t* is greater than 600, the growth rates tend to flatten out. In the decoding delay region of [100, 1000], the message update reduction ratio of PJEWD to JEWD increases from 26% to 48%; while the reduction rate of the proposed JHWD to JEWD increases from 58% to 94%. In other words, the message updates reduction ratio of the proposed JHWD is about two times higher than the PJEWD algorithm.

**Figure 4:**
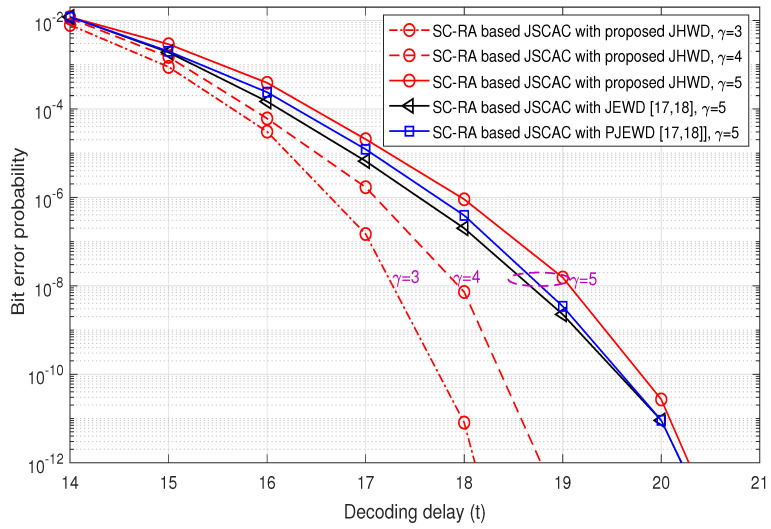
Asymptotic anytime property analysis based on the density evolution [17,18].

**Figure 5:**
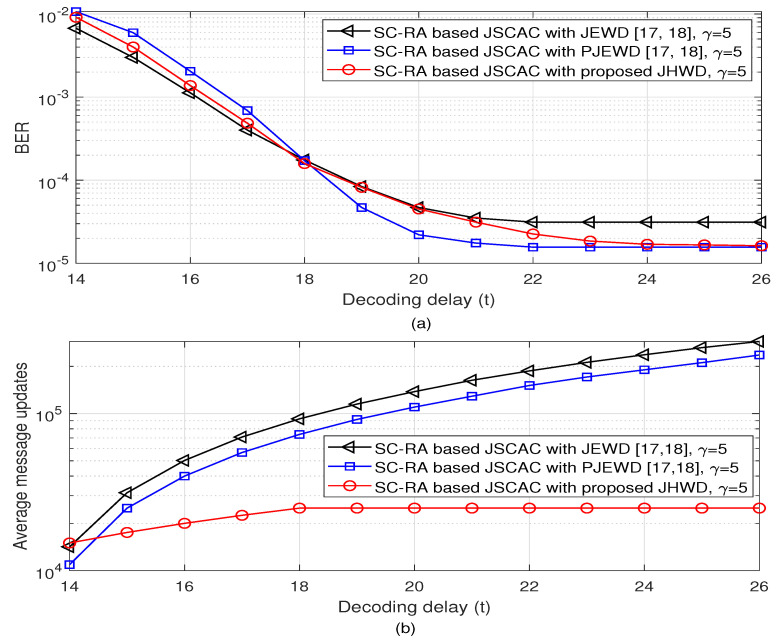
BER performance and decoding complexity of a single message block with $N^{sc}=64$ bits as the decoding delay within a sliding window region [17,18].

In Figure 4, the asymptotic anytime properties of SC-RA-based JSCAC with different decoding schemes are analyzed. The anytime properties of a single block in the first sliding window region is observed from t=14. It can be seen that with the same coupling length of γ=5, the bit error probability curves of the JEWD and PJEWD algorithm have similar descent rates, while the curve slope of the proposed JHWD algorithm is slightly slower. Figure 4 also shows the error probabilities of the proposed JHWD with different γ. It can be seen that the smaller the coupling length, the larger the curve slope. However, in the finite length regime (as seen in Figure 5), a smaller coupling length tends to lead a higher error floor. Correspondingly, as shown in Figure 3, the greater the coupling length, the higher the decoding complexity. These results indicate that the anytime property and decoding complexity of the proposed JHWD are related to the coupling length γ, and a proper coupling length should be selected to trade off the error performance and decoding complexity.

In Figure 5, the simulation performances of a single message block is observed as a function of decoding delay within the first sliding window region, from t=14, with a source block length of $N^{sc}=64$ bits and $E_{b}/N_{0}$ is 2 dB. As shown in Figure 5a, the bit error rate (BER) curve of the proposed JHWD algorithm is located between the JEWD and PJEWD algorithms, which is consistent with the numerical results in Figure 4; and it lastly reaches the error floor at t=26 (also within the sliding window size of 3γ−2). Moreover, the error floor of the proposed JHWD algorithm is closer to that of the PJEWD algorithm. When the coupling length is larger, i.e., γ=8, the slops of the BER curves are slightly smaller than those of γ=5 before the time slot t=5, but can achieve lower BER performance. when t=21, the BER of the proposed JHWD algorithm is lower than $10^{-6}$. Figure 5b shows the average message updates of a single message block as the decoding delay. The message updates of the JEWD and PJEWD algorithms approximately linearly increase within the observed time slot region. As to the hybrid window decoding of the proposed JHWD algorithm, the adaptive local expanding window decoding strategy with smaller iteration rounds of $I_{\max2}$ does not result in a significant increase of message updates, which is about 10 times lower than those of the JEWD and PJEWD algorithms at t=26. When the coupling length is γ=8, the average message updates of the proposed JHWD algorithm are relatively higher than those of γ=5. This experiment indicates that the anytime property of the proposed JHWD algorithm is between the JEWD algorithm and PJEWD algorithm but with lower decoding complexity.

**Figure 6:**
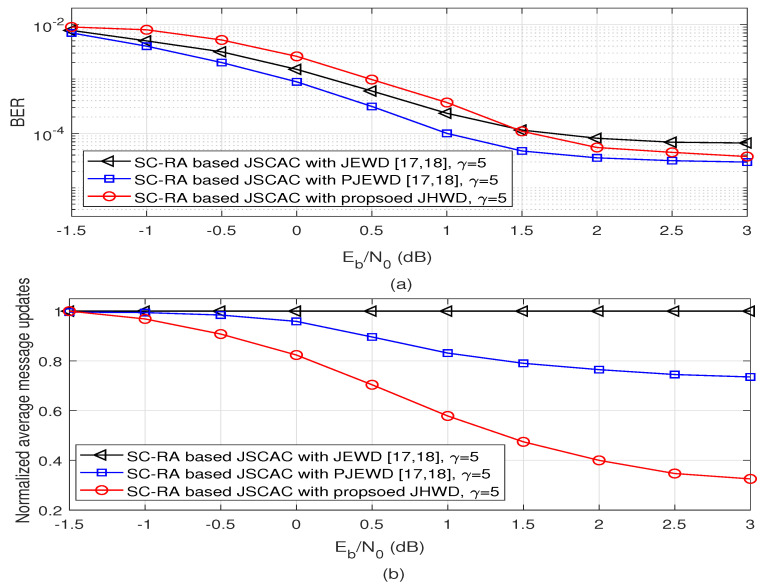
BER performance and decoding complexity of the data chain at the time slot t=100 (L = 100) with $N^{sc}=64$ bits [17,18].

In Figure 6, the simulation performance of the data chain at the time slot *t* = 100 versus $E_{b}/N_{0}$ is observed. In Figure 6a, the proposed JHWD algorithm has the smallest curve slope in the low signal to noise ratio (SNR) region. In the high SNR region, larger than 1.5 dB, the proposed JHWD algorithm can obtain a lower error floor than that of JEWD algorithm and is closer to the BER performance of the PJEWD algorithm. Figure 6b shows the average message updates normalized by that of the JEWD scheme. The decoding complexity of traditional JEWD does not change with SNR. While the decoding complexities of the proposed JHWD and PJEWD scheme are lower than that of the JEWD scheme, especially in the high SNR region. When $E_{b}/N_{0}$ = 3 dB, the average message updates of PJEWD can be decreased about 26.47%, while the proposed JHWD can be decreased about 69.61% compared with the JEWD algorithm. These simulation results show that the proposed JHWD can reduce the decoding complexity to a great extent without sacrificing too much error correction performance.

**Figure 7:**
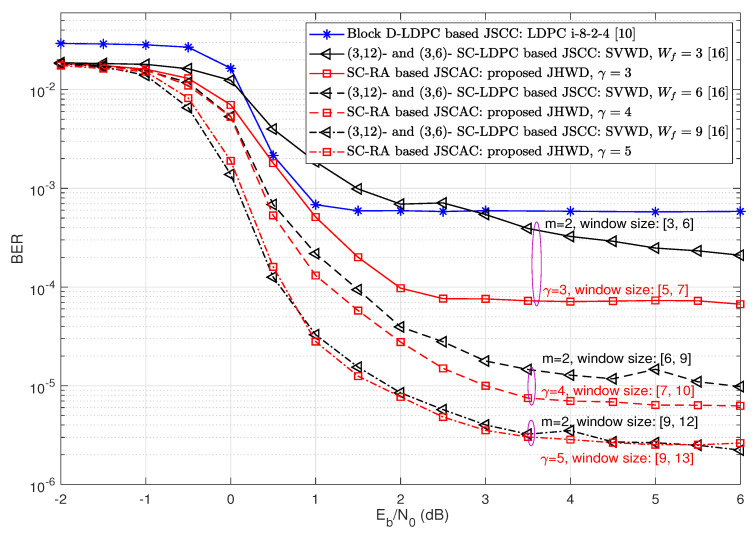
BER performance comparisons with other kinds of JSCC schemes, $R^{sc}=0.25$, $R^{cc}=0.5$, $N^{sc}=640$ bits, $N^{cc}=320$ bits, and L=20 [10,16].

Figure 7 provides the BER performance comparisons with the state of the art, especially the (3,12)- and (3,6)- SC-LDPC-based JSCC scheme with sliding variable-window decoding (SVWD) of comparable window size [16]. As seen in Figure 7, with the same code rates of $R^{sc}$ = 0.25, $R^{cc}$ = 0.5 and the code length of 6400 bits, the block D-LDPC-based JSCC (LDPC i-8-2-4) [10] has the highest error floor. When the coupling length γ is 3, which is comparable to the syndrome former memory of m=2 in [16] (γ=m+1), and the adaptive decoding window size is 2γ−1=5 to 3γ−2=7 (versus the sliding variable-window size of $W_{f}=3$ to $W_{max}=6$ in [16]), the SC-RA-based JSCAC with proposed JHWD has a lower error floor. However, as γ is increased to match the larger window sizes of [16], the preponderance of SC-RA-based JSCAC with the proposed JHWD is gradually reduced. When γ is 5, the JSCAC scheme with the proposed JHWD demonstrates a similar performance to the SC-LDPC JSCC scheme with SVWD of $W_{f}=9$. The narrowing gap might be caused by the mismatch between γ and *m* in [16].

## 5. Conclusions

This paper considers a decoding scheme to reduce the decoding complexity without compromising the error correction performance for JSCAC system. We have observed that the improvement of post-decoding BER of the global expanding window decoding in a JSCAC system with finite coupling length gradually decreases with the decoding delay, while the decoding complexity increases approximately linearly. Our key idea, called joint hybrid window decoding (JHWD), is to implement an adaptive local expanding window decoding within the sliding window region. We have also proposed an improved density evolution algorithm to analyze the asymptotic decoding complexity of the JHWD algorithm. The numerical analyses and simulation results have shown that the proposed JHWD algorithm can greatly reduce the decoding complexity while maintaining BER performance similar to that of prior-art decoding schemes, which could be a good alternative for the application of JSCAC systems in low complexity scenarios.

## Data Availability

The data presented in this study are available on request from the corresponding author.

## Abbreviations

The following abbreviations are used in this manuscript:

AWGN	additive white Gaussian noise
BER	bit error rate
BP	belief propagation
BPSK	binary phase-shift keying
CNs	check nodes
CSI	channel state information
D-LDPC	double low-density parity check
DP-LDPC	double protograph LDPC
JEWD	joint expanding window decoding
JHWD	joint hybrid window decoding

JSCC	joint source-channel coding
JSCAC	joint source-channel anytime coding
LLRs	log-likelihood ratios
PJEWD	partial joint expanding window decoding
PNs	parity bit nodes
INs	information bit nodes
SNR	signal to noise ratio
SNs	syndrome nodes
SC-LDPC	spatially coupled LDPC code
SSI	source significance information
SC-RA	spatially coupled repeat-accumulate code
UEP	unequal error protection
